# High-Throughput Method for the Simultaneous Determination of Doxorubicin Metabolites in Rat Urine after Treatment with Different Drug Nanoformulations

**DOI:** 10.3390/molecules27041177

**Published:** 2022-02-09

**Authors:** Lara Zorić, Nikša Drinković, Vedran Micek, Leo Frkanec, Akif Emre Türeli, Nazende Günday-Türeli, Ivana Vinković Vrček, Ruža Frkanec

**Affiliations:** 1 Faculty of Pharmacy and Biochemistry, University of Zagreb, Ante Kovačića 1, 10000 Zagreb, Croatia; larazoric488@gmail.com; 2 Poliklinika Prof. Nikša Drinković, Boškovićeva Ul. 15, 10000 Zagreb, Croatia; ndrink@gmail.com; 3 Institute for Medical Research and Occupational Health, Ksaverska Cesta 2, 10000 Zagreb, Croatia; vmicek@imi.hr; 4 Rudjer Boskovic Institute, Bijenička Cesta 54, 10000 Zagreb, Croatia; frkanec@irb.hr; 5 MyBiotech GmbH, Industriestraße 1B, 66802 Überherrn, Germany; e.tuereli@mybiotech.de (A.E.T.); n.guenday-tuereli@mybiotech.de (N.G.-T.); 6 Centre for Research and Knowledge Transfer in Biotechnology, University of Zagreb, Rockefellerova 10, 10000 Zagreb, Croatia

**Keywords:** high-throughput analysis, doxorubicin metabolites, HPLC-FD method, validation, nanoformulation

## Abstract

Doxorubicin (DOX) is one of the most effective cytotoxic agents against malignant diseases. However, the clinical application of DOX is limited, due to dose-related toxicity. The development of DOX nanoformulations that significantly reduce its toxicity and affect the metabolic pathway of the drug requires improved methods for the quantitative determination of DOX metabolites with high specificity and sensitivity. This study aimed to develop a high-throughput method based on high-performance liquid chromatography with fluorescence detection (HPLC-FD) for the quantification of DOX and its metabolites in the urine of laboratory animals after treatment with different DOX nanoformulations. The developed method was validated by examining its specificity and selectivity, linearity, accuracy, precision, limit of detection, and limit of quantification. The DOX and its metabolites, doxorubicinol (DOXol) and doxorubicinone (DOXon), were successfully separated and quantified using idarubicin (IDA) as an internal standard (IS). The linearity was obtained over a concentration range of 0.05–1.6 μg/mL. The lowest limit of detection and limit of quantitation were obtained for DOXon at 5.0 ng/mL and 15.0 ng/mL, respectively. For each level of quality control (QC) samples, the inter- and intra-assay precision was less than 5%. The accuracy was in the range of 95.08–104.69%, indicating acceptable accuracy and precision of the developed method. The method was applied to the quantitative determination of DOX and its metabolites in the urine of rats treated by novel nanoformulated poly(lactic-co-glycolic acid) (DOX-PLGA), and compared with a commercially available DOX solution for injection (DOX-IN) and liposomal-DOX (DOX-MY).

## 1. Introduction

**Figure 1:**
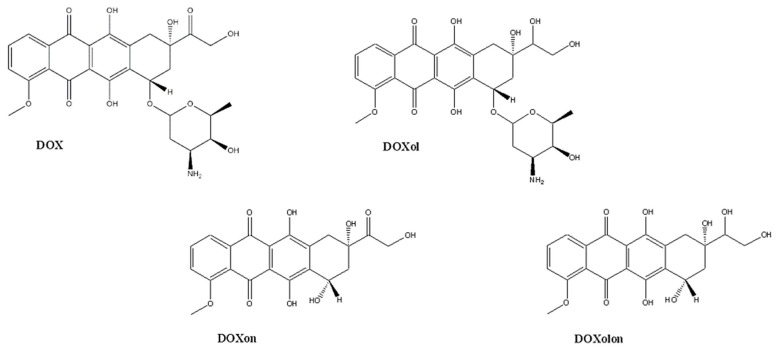
Chemical structures of doxorubicin and its metabolites.

The treatment of malignant diseases poses many challenges that need to be overcome, due to their wide prevalence and high mortality rates [1]. Anthracyclines are a group of antitumor drugs that were isolated from the genus Streptomyces in the 1960s, and their main representatives are doxorubicin (DOX, depicted in Figure 1) and daunorubicin [2]. They are used in the treatment of numerous solid tumors and hematological malignancies, including leukemia, lymphoma, breast, and ovarian tumors [3,4]. Anthracyclines are generally subject to hepatic metabolism. DOX enters cells by free diffusion, and achieves its action in the nucleus and mitochondria. The three major DOX metabolites are produced in cells (Figure 1), i.e., doxorubicinol (DOXol), doxorubicinone (DOXon), and doxorubicinolone (DOXolone) [5]. According to the available data, almost 50% of unchanged DOX, 30% of DOXol, and 9% of the DOX aglycone, including DOXon, are excreted in the urine [6]. Animal studies have demonstrated that each of these metabolites is formed under NADPH-dependent conditions, suggesting the existence of specific enzymes involved in DOX metabolism [7,8]. For pharmacokinetic and pharmacodynamics studies, the determination of DOX, DOXol, and DOXon in urine, plasma, liver, spleen, and heart tissues is of clinical relevance [9,10]. The most significant role in the induction of cardiotoxicity has been ascribed to DOX and DOXol, but the exact mechanism of their toxic action has not been fully elucidated. DOXol is more hydrophilic than DOX and enters cardiomyocytes to a greater extent, causing more damage [6]. Furthermore, DOXone or DOX hydroxyaglycone is formed by hydrolysis of the glycosidic bond of DOX, while further carbonyl reduction of DOXon produces DOXolone. In the heart, the amount of DOXon is negligible, as it is almost immediately converted to DOXolone [7]. Even though significantly less DOXon is produced by metabolism compared to DOXol, measurable and clinically significant amounts of this metabolite are still found in urine.

Despite being one of the most commonly and widely used antitumor agents, the efficacy of DOX is jeopardized by undesired side effects, such as cardiotoxicity, systemic organ toxicity, and inflammation, as well as the development of tumor resistance [4]. Another important problem of DOX is its low selectivity and inability for targeted delivery to the diseased site [11]. Nanotechnology may significantly advance antitumor therapy by using nano-enabled carriers, with the aim of improving drug solubility, stability, pharmacokinetics, and pharmacodynamics (PK/PD) properties [12,13]. One of the clinically approved DOX nanoformulations is the liposomal nanoformulation. Liposomes have been identified as suitable drug delivery systems in various therapeutic areas, and, due to their non-toxicity, non-immunogenicity, and biodegradability, liposomes are completely physiologically acceptable [14,15]. The incorporation of DOX into liposomes increases its elimination half-life (t½) and distribution in tumor tissues; for example, Myocet liposomal (Teva B. V., Jerusalem, Israel) is a liposomal DOX formulation approved by the US Food and Drug Administration (FDA) and European Medicines Agency (EMA) for the treatment of metastatic breast and ovarian cancer. Another type of nano-drug delivery system is a nanoformulation based on polylactic-co-glycolic acid (PLGA), a copolymer of lactic and glycolic acid, that is biodegradable and hydrolyzed in the body to lactic and glycolic acid [16]. PLGA has been approved by the FDA as a carrier of numerous drugs, including DOX, and it offers many advantages in cancer therapy, as it enters the capillaries through the circulation and is passively directed into the tumor tissue, due to increased permeability and retention in the capillaries of tumor tissue. Various techniques for the incorporation of DOX into PLGA nanoparticles have been described [17,18], and the slow DOX release from such nanoformulations confirms its high potential for clinical use [19].

Despite numerous studies describing bioanalytical methods for testing the efficacy and PK/PD profile of DOX, a very limited number of HPLC-FD-based methods have been reported for the simultaneous quantitative analysis of DOX and its metabolites in urine, without prior sample preparation [20,21,22,23]. The main goal of this work was to develop and validate a simple, fast, highly sensitive, and selective bioanalytical method that allows the high-throughput quantitative analysis of DOX and its metabolites in small volumes of urine samples. The specific objectives were to carry out each individual step in the validation of the method, according to the recommendations described in the Technical Requirements for Pharmaceuticals for Human Use of the International Council for Harmonisation (ICH). Thus, the development of the method was focused on ensuring that all the parameters for each individual compound were within the given limits. The method was validated by examining the specificity and selectivity, linearity, accuracy, precision, limit of detection (LOD), and limit of quantification (LOQ). Finally, the developed and validated method was used to compare a sustained release and metabolic degradation of novel DOX-PLGA formulation with commercially available and clinically approved Myocet liposomal (DOX-MY) and DOX solution for injection (DOX-IN). Urine samples of male and female Wistar rats treated with these three DOX formulations were analyzed following a clinically relevant administration approach.

## 2. Materials and Methods

### 2.1. Reagents

DOX, DOXol, DOXon and idarubicin (IDA) internal standards (IS) were purchased from Toronto Research Chemicals (Toronto, Ontario, QC, Canada). Trichloro-acetic acid (TCA) was provided by Sigma-Aldrich (St. Louis, MI, USA). Acetonitrile (ACN) (HPLC grade), methanol (HPLC grade), and dimethyl sulfoxide (DMSO) were purchased from Merck (Darmstadt, Germany).

For the animal experiments, DOX-IN (2 mg/mL) was purchased from PLIVA pharmaceutical company (Zagreb, Croatia), DOX-MY formulation containing 2 mg/mL of DOX was purchased from Teva B. V. Company (Jerusalem, Israel), and DOX-loaded PLGA nanoparticles (DOX-PLGA) containing 4 mg/mL of DOX were designed and produced by MyBiotech GmbH (Überherrn, Germany).

### 2.2. HPLC Instrumentation and Conditions

The HPLC system (Waters, Milford, CT, USA) consisted of an In-line Degasser AF, 600 Pump, 2479 Fluorescent Detector, 717 pulse Autosampler and a computer with the HPLC program Empower. Reversed-phase separation was performed on an Atlantis dC18 3μm 4.6 × 150 mm analytical column, provided by Waters. The mobile phase, consisting of ACN and water with 0.05% TCA (65/35, *v*/*v*), was delivered isocratically at a flow rate of 1 mL/min. Fluorescence detection was carried out at an excitation wavelength (λ_ex_) of 480 nm and an emission wavelength (λ_em_) of 558 nm. The column temperature was maintained at 29 °C and the injection volume was 10 µL.

### 2.3. Preparation of Standards and Quality Control Samples

Solutions of DOX, DOXol, DOXon and IDA were prepared by dissolving 1 mg of each compound in 0.5 mL of DMSO, and they were stored at 4 °C until use. Stock solutions (100 µg/mL) of each standard were prepared by separately diluting initial solutions (2 mg/mL) in methanol. Solutions in the concentration range 0.005–5 µg/mL were obtained by further dilution of stock solutions in methanol, and were stored at −20 °C until analysis was performed.

Quality control samples (QCs) were prepared by spiking urine samples of control (untreated) male and female Wistar rats (CS, control samples) with a stock solution of DOX and each tested metabolite (DOXol and DOXon). Final six concentrations for each compound ranged between 0.05 and 1.6 µg/mL (0.05, 0.1, 0.2, 0.4, 0.8 and 1.6 µg/mL) and were also stored at –20 °C to reduce the level of degradation of DOX and its metabolites. Before HPLC analysis, QCs were filtered on 0.45 µm filter (Millipore, Burlington, VT, USA) prior to addition of IS. The concentration of IS in every QCs was 1 µg/mL.

### 2.4. In Vivo Experiment

Animal experiments were performed on 3-month-old Wistar male and female rats, bred in the Animal Facility Unit of the Institute for Medical Research and Occupational Health (Zagreb). Testing was performed in accordance with the Animal Protection Act (OG 135/06) and the Ordinance on the protection of animals used for experimental and other scientific purposes (OG, 47/11). The whole study was approved by the Ethical Committee of the Institute for Medical Research and Occupational Health (protocol title: “Učinkovitost i sigurnost primjene nanoformulacije za smanjenje kardiotoksičnosti doksorubicina”, protocol number: 100-21/19-24) and by the Directorate of Veterinary and Food Safety of the Ministry of Agriculture of the Republic of Croatia (protocol title: “Primjena nanoformulacija za smanjenje kardiotoksičnosti doksorubicina”, approval number: P/8634610).

A developed and validated method for the simultaneous determination of DOX and its metabolites was used in the urine analysis of animals that were treated with three different DOX formulations. Rats were divided into 4 groups with 8 rats in each, for both male and female rats. The first experimental group consisted of control animals, the second experimental group was treated with DOX-IN, the third group received DOX-MY, and the last group received DOX-PLGA.

Rats were injected intraperitoneally with one dose (3 mg of DOX equivalent/kg of body weight) every 6 days for a total of 4 doses each over 24 days. Prior to each administration, the animals were kept in a metabolic cage for 24 h. Urine was collected 12 h after application and stored at −20 °C immediately after collection. Prior to the HPLC analysis, the urine samples were filtered through 0.45 μm filters (Millipore). In each urine sample, creatinine concentration was determined with a rate-blanked creatinine/Jaffé method using an automated Hitachi 917 analysis system (Roche Diagnostics, Mannheim, Germany). These concentrations were later used to normalize data for DOX, DOXol, and DOXon to urinary creatinine concentration, as the time of urine collection, urine concentration, and urine flow rate may significantly affect the concentration of urine biomarkers.

The IS was added to each urine sample at the concentration of 1 μg/mL. After determination of DOX, DOXol, and DOXon urinary concentrations, these data were normalized to urinary creatinine concentrations and expressed as mg of tested compound per g of creatinine. All data were given as mean values of concentrations found in urine samples of 8 animals, with their respective standard deviations. Differences in DOX, DOXol, and DOXon urinary concentrations between males and females were tested using a Mann–Whitney U-test, using the Statistica Software 13.5.0.17 (TIBCO Software Inc., Palo Alto, CA, USA). The minimal significance level was *p* < 0.05.

## 3. Results

### 3.1. Validation of the HPLC-FD Method

#### 3.1.1. Specificity and Selectivity

**Figure 2:**
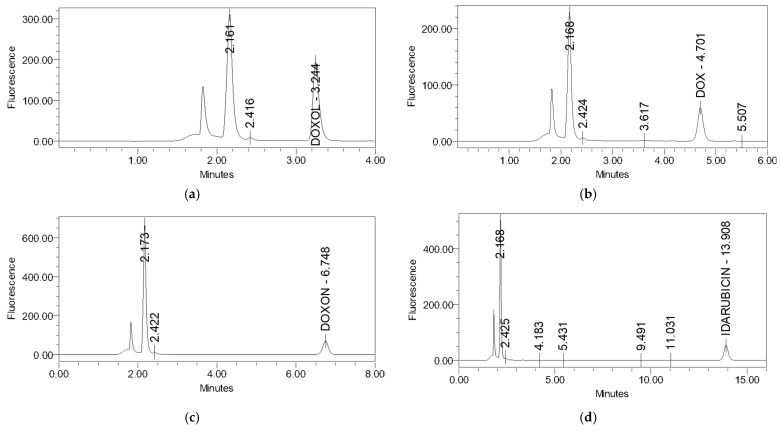
Standards of DOX metabolites and IS in control urine (1 µg/mL): (**a**) DOXol; (**b**) DOX; (**c**) DOXon; (**d**) IDA.

**Figure 3:**
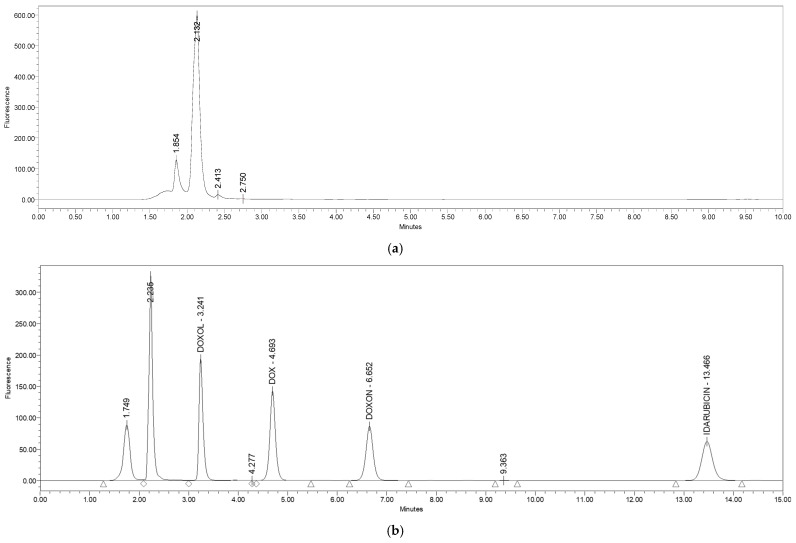
Chromatograms of (**a**) blank urine sample; (**b**) a mixture of standards: DOX, DOXol, and DOXon in control urine sample (concentration 1 µg/mL) with addition of IS (1 µg/mL).

The selectivity and specificity of the method, shown in Figure 2, were tested by spiking the urine of untreated CS rats with standard solutions of DOXol, DOX, DOXon, and IDA, giving the final concentration of 1 µg/mL for each metabolite. Figure 3 represents the chromatogram of all the standards (1 µg/mL) in the control urine.

The figures show that every standard has different retention times, i.e., the chromatographic maximums do not overlap, confirming the specificity and selectivity of the method. The analyte retention times are 3.243 min for DOXol, 4.700 min for DOX, 6.639 min for DOXon, and 13.909 min for IDA. The deviation in retention time was negligible in repeated analyses in hexaplicate for all three analytes (ranging between ±0.001 min and ±0.002 min).

Additionally, the analysis conditions were optimally adjusted, and the separation of all four compounds (DOX, DOXol, DOXon, IDA) was complete and no interference with urine compounds was detected. Excellent resolution (greater than one for every analyzed compound) and complete separation of the analyzed compounds were achieved by the selected and applied analytical column and stationary phase used in the described method.

#### 3.1.2. Linearity

The linearity of the method was determined by analyzing the following six concentration levels of standards: 0.05, 0.10, 0.20, 0.40, 0.80, and 1.60 µg/mL. The solutions were injected three times. The mean value of the area below the chromatographic maximum, the regression direction equation, and the correlation coefficient were calculated as shown in Table 1.

The correlation coefficient fulfills the set criterion, since it is greater than 0.9995 for each metabolite. The obtained results are consistent with the results described in [9].

#### 3.1.3. Precision

The precision of the method is expressed by the relative standard deviation (RSD %) of the replicate measurements as a measure of random error. The intra-day precision was determined by analyzing the QC samples. The intra-day and inter-day precisions, shown in Table 2, were determined by analyzing six replicates of the QC samples three times over three days.

The RSD ranges from 0.03% for DOX (intra-day precision, second day) to 4.83% for DOXol (inter-day precision), which is within the set criteria of 5% for the precision of the HPLC method [24].

#### 3.1.4. Accuracy

Accuracy is the degree of compliance between the actual, reference, and experimental values. It was determined in triplicate for at least three different concentrations. The results are presented as a recovery value, which was calculated as a ratio of the theoretical and measured values. Since IS was used, the internal response factor (IRF) was determined for DOX, DOXol, and DOXon.

Three different concentrations for each metabolite (0.50, 0.75, and 1.00 µg/mL) were used for the determination of accuracy. The samples were prepared by spiking CS urine samples. The results, shown in Table 3, are expressed as recovery values with the corresponding RSD values.

The parameters of accuracy confirmed that the developed method is within acceptable limits. The recovery values ranged from 95.08% for DOXon in urine spiked with 1.00 µg/mL DOXon to 104.69% for DOX in urine spiked with the same DOX concentration.

#### 3.1.5. Limit of Detection (LOD) and Limit of Quantification (LOQ)

The lowest detectable concentration (LOD) and the lowest concentration that can be accurately and precisely determined (LOQ) can be defined by statistical analysis, based on the deviation signal and the slope. The slope was calculated from a calibration curve and the standard deviation was defined as the residual standard deviation of linear regression. The parameters that were calculated and used for the determination of LOD and LOQ are shown in Table 4.

Our results show that the method is highly sensitive for the determination of DOX and its metabolites, especially for DOXon. For DOXon, the LOD was 0.005 μg/mL and the LOQ was 0.015 μg/mL.

### 3.2. Quantitative Analysis of DOX and Its Metabolites in Urine of Rats Treated with Different DOX Formulations

**Figure 4:**
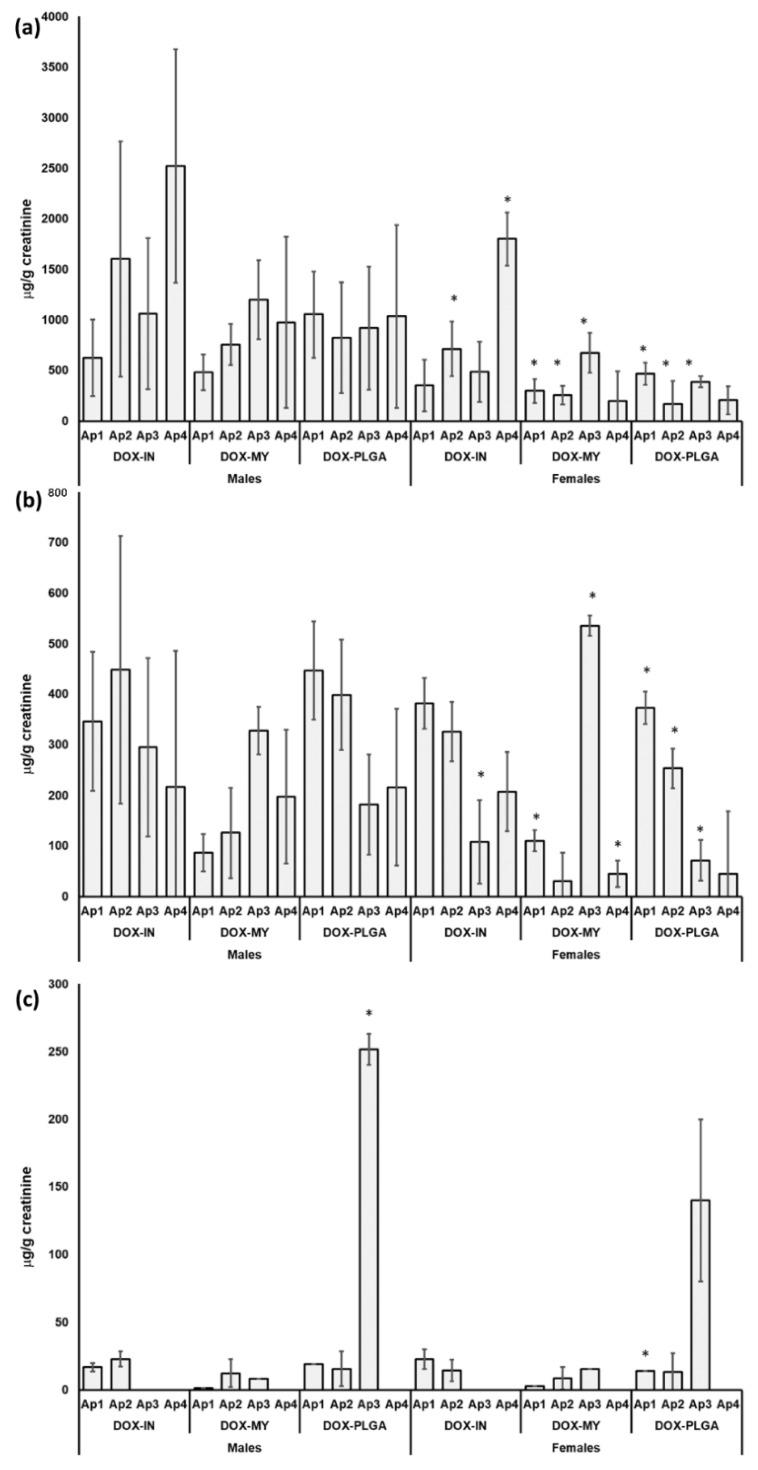
The concentrations of (**a**) doxorubicin (DOX) and its metabolites; (**b**) doxorubicinol (DOXol); (**c**) doxorubiconone (DOXon) in the urine of male and female Wistar rats after each of 4 intraperitoneal applications (Ap) of conventional DOX solution for injection (DOX-IN), a commercial liposomal DOX formulation (DOX-MY) and DOX-loaded PLGA nanoparticles (DOX-PLGA). Results are normalized by the concentration of creatinine in the urine, expressed as µg/g of creatinine and shown as mean values of concentrations found in urine samples of 8 animals, with their respective standard deviations (error bars). Significant differences in the concentration of each tested substance after each application in urine of females compared to males are denoted with * (at *p* < 0.05).

The concentrations of DOX and its metabolites found in the urine of male and female Wistar rats after treatment with DOX-IN, DOX-MY, and DOX-PLGA are presented in Figure 4.

In the urine of all the treated animals of both sexes, DOX and DOXol were detected, while DOXon was only found sporadically and at much lower concentrations compared to DOX and DOXol. The highest concentrations were found for excreted DOX in the groups treated with DOX-IN. Females showed significantly lower levels of all the tested analytes. A discrete difference in DOX and DOXol excretion between the DOX-MY and DOX-PLGA groups was observed. The total cumulative amount of unchanged DOX excreted in urine was much higher in the animals treated with DOX-IN, compared to the other two formulations.

## 4. Discussion

Nano-delivery systems minimize the toxicity and increase the bioavailability of DOX, preventing its degradation in circulation [25,26]. Due to their structural features and biological properties, biodegradability, biocompatibility, non-immunogenicity, and non-toxicity, liposomes are physiologically acceptable and can be used in different therapeutic areas. Liposomes can modify the pharmacokinetic properties of encapsulated DOX, enhance its bioavailability, and reduce the systemic side effects and toxicity [27,28]. The current successful and clinically approved lipid-based formulations of DOX include non-pegylated (Myocet) and pegylated (Doxil^®^) formulations. PLGA nanoparticles represent another attractive drug carrier, due to the biodegradability of PLGA towards non-toxic metabolites, such as lactic acid and glycolic acid, upon hydrolysis in the body [29]. This study describes a novel and simple HPLC-FD-based method for the high-throughput simultaneous determination of DOX and its metabolites in urine after treatment with different DOX nanoformulations. Every individual step in the validation of the method was performed according to the ICH recommendations (ICH Topic Q 2 (R1) Validation of Analytical Procedures: Text and Methodology, CPMP/ICH/381/95) [30].

Although different analytical techniques to quantify the anthracycline levels in urine or serum have been reported in the literature [31,32], the developed method has been improved with respect to the sample preparation and analysis conditions. No extraction procedures before analysis are required, and complete chromatographic separation was accomplished using a mobile phase, without buffer and salt only. The mobile phase based on ACN and water, with TCA as an ion-pairing regent, ensures longer column usage and avoids the precipitation of salt on the column. The simultaneous determination of doxorubicin and its metabolites DOXol and DOXon, in a small volume of urine, was performed on an inexpensive and easily accessible Cl8 reversed-phase column, with a short run time and with excellent precision and accuracy. The method was fully validated by examining its specificity and selectivity, linearity, accuracy, precision, limit of detection, and limit of quantification. It also meets all the validation criteria for each individual compound. This is a great advantage because of how demanding and time consuming the validation process is. The method can be easily used in other cases, with an appropriate plan for partial validation and cross-validation. The developed method is characterized by high selectivity and specificity (Figure 2). The chromatogram of all the standards in the urine of the control group (Figure 3) shows that all three working standards have different retention times and the chromatographic maxima do not overlap. There were no interferences with the urine matrix under the prescribed test conditions, while the deviation in retention time was negligible (±0.001–0.002 min) for all three analytes. The resolution was higher than one for all the analyzed compounds and complete separation was achieved. The test of linearity was performed at six concentration levels. The results are presented graphically (Figures S1–S3, Supplementary Materials) and in tabular form (Table S1) for each metabolite. The correlation coefficient for each metabolite was calculated, and linearity in the tested concentration range was confirmed.

The intra-test and inter-test accuracy for all three metabolites and IS were verified. The inter-test accuracy, or matching of the results obtained by measuring the same sample over a longer period, was determined in three separate tests performed in hexaplicate over three days, showing an RSD range from 0.03% for DOX within intra-test accuracy for 2 days to 4.83% for DOXol within inter-test accuracy, with 5% for precision (Table 2). Testing the accuracy of the method showed that the analytical yield or recovery ranged from 95.08% for DOXon to 104.69% for DOX in urine spiked with 1 µg/mL of analytes (Table 3), thus meeting the required criteria. The detection limit (LOD) and the quantification limit (LOQ) were calculated, and the obtained results (Table 4) confirmed that the method is highly sensitive for the simultaneous determination of DOX and its metabolites in rat urine. High precision was achieved due to the fluorescence detection applied, which is highly sensitive and widely used in the development of a bioanalytical method [33,34].

The obtained levels of DOX and its metabolites in the urine samples of male and female rats (Figure 4) evidenced that treatment with nanoformulations, DOX-MY, and DOX-PLGA led to a lower amount of excreted DOX after each application in both males and females. Significant differences in the urine concentrations of DOX, DOXol, and DOXin between males and females (Figure 4) indicated sex-related differences in the metabolism and excretion of DOX, which should be mechanistically investigated. Thus, future efforts should be directed towards the elucidation of how sex could affect the pharmacokinetic and pharmacodynamic profile of different drug formulations. Females showed lower concentrations of unchanged, excreted DOX compared to males [35,36]. These results are indicative of higher retention and sustained release of DOX in the body after treatment with the nanoformulations. Moreover, the highest DOX concentration was found in the urine of DOX-IN-treated animals after the last injection, which implies the higher organ toxicity of the conventional DOX formulation compared to the nanoformulation. As DOX is mostly metabolized by the liver, the higher excretion of unchanged DOX means a lower capacity of the liver to metabolize it [37,38]. This was also evident in the results obtained for DOXol and DOXon concentrations, which decreased with the number of applications in animals treated with DOX-IN. This was not observed in animals treated with DOX-MY and DOX-PLGA. Moreover, DOXon was not found in the urine after the third and fourth applications of DOX-IN, while its highest amount in the urine was observed in both male and female rats after the third application of DOX-PLGA, which was accompanied by the lowest DOX amount. This observation may indicate greater uptake and metabolism efficiency of DOX-PLGA by the liver, in comparison to the DOX uptake efficiency. However, the higher excretion of DOX after treatment with DOX-IN, compared to DOX-MY and DOX-PLGA, may also be due to the larger molecular weight and higher lipophilicity of nanoformulations compared to conventional formulations that decreased their filtration by the glomerulus. Accurate information about the absorption, distribution, metabolism, and excretion profile of each DOX formulation is not possible to provide without obtaining data on the DOX pharmacokinetic profile in serum and its accumulation in different organs, which was beyond the scope of this study.

## 5. Conclusions

In the present study, a high-throughput HPLC-FD method for the complete separation and simultaneous quantitative determination of unchanged DOX and its metabolites, DOXol and DOXon, in rat urine was described. Separation was achieved with a Cl8 reversed-phase column and acetonitrile/water as a mobile phase, with TFA as an ion-pairing reagent. The urine samples were analyzed without prior purification. The developed and validated method, which had high sensitivity, selectivity, and accuracy, has been successfully applied for the determination of DOX and its metabolites in rat urine treated with different DOX formulations. The method was validated according to the ICH guidelines for all three analyzed compounds, and showed high selectivity, linearity (k ≥ 0.999), accuracy (95% ≤ analytical yield ≤ 105%), and precision (RSD < 5%) in the working area for each individual compound. The advantages of using idarubicin as an internal standard in quantitative determination are visible in the high accuracy and precision of compound determination and inter- and intra-test precision results. It was shown that the urine samples of animals treated with the liposomal nanoformulation DOX-MY had a significantly smaller amount of DOX and its metabolites compared to the other two groups, while the animals treated with DOX-PLGA showed the highest amount of the DOX-on metabolite. The obtained results highlight the effect of the DOX formulation on its metabolic pathway.

## Figures and Tables

**Table 1:** Parameters of linear regression.

Metabolite	Area of Linearity (µg/mL)	Regression Equation	Correlation Coefficient (R^2^)
DOX	0.05–1.60	y = 515,381x − 17,769	0.9996
DOXol	0.05–1.60	y = 10^6^x − 17,839	0.9996
DOXon	0.05–1.60	y = 803,820x − 848	0.9999

**Table 2:** Intra- and inter-day precision.

Metabolite	Parameter of Precision	Intra-Day Precision			Inter-Day Precision
		1st day	2nd day	3rd day	
DOX (1 µg/mL)	*x_avg_*:	457,794.60	464,999.60	465,950.5	462,914.90
	*S_d_*:	1353.18	1508.11	1210.58	4459.73
	RSD (%):	0.30	0.03	0.26	0.96
DOXol (1 µg/mL)	*x_avg_*:	1,023,770.00	1,088,522.00	1,126,937.00	1,079,743.00
	*S_d_*:	1859.63	3112.16	5250.95	52,140.97
	RSD (%):	0.18	0.29	0.47	4.83
DOXon (1 µg/mL)	*x_avg_*:	711,946.10	747,576.10	752,071.60	737,197.90
	*S_d_*:	2091.75	1513.79	2837.71	21,983.94
	RSD (%):	0.29	0.20	0.38	2.98
IDA (1 µg/mL)	*x_avg_*:	836,266.60	807,980.40	854,275.10	832,840.70
	*S_d_*:	1957.55	4042.85	17,706.24	23,336.72
	RSD (%):	0.23	0.50	2.07	2.80

**Table 3:** Parameters of accuracy.

		0.50 µg/mL		0.75 µg/mL		1.00 µg/mL	
	IRF	Recovery (%)	RSD (%)	Recovery (%)	RSD (%)	Recovery (%)	RSD (%)
DOX	0.88	102.05	0.36	99.04	0.16	104.69	0.00
DOXol	0.86	102.73	1.28	100.61	0.69	97.73	2.48
DOXon	1.01	104.68	0.4	101.13	4.99	95.08	0.24

**Table 4:** Calculated parameters for the determination of LOD and LOQ.

Metabolite	*S_e_*	*S_d_*	LOD (μg/mL)	LOQ (μg/mL)
DOX	3852.24	1572.67	0.010	0.031
DOXol	8594.37	21,051.83	0.007	0.021
DOXon	2875.77	1174.03	0.005	0.015

## Data Availability

Not applicable.

## Supplementary Materials

The following supporting information can be downloaded online. Figure S1: Calibration curve for DOX; Figure S2: calibration curve for DOXon; Figure S3: calibration curve for DOXol; Table S1: Parameters of linearity; Concentration of doxorubicin (DOX) and its metabolites doxorubicinol (DOXol) and doxorubiconone (DOXon) in urine of female Wistar rats after each of 4 intraperitoneal applications of DOX-IN, DOX-MY and DOX-PLGA; Table S2: Concentration of doxorubicin (DOX) and its metabolites doxorubicinol (DOXol) and doxorubicinone (DOXon) in urine of female Wistar rats after each of 4 intraperitoneal applications of DOX-IN, DOX-MY and DOX-PLGA; Table S3: concentrations of doxorubicin metabolites determined in male mice 12 h after injection on four consecutive days; Table S4: Average concentrations of doxorubicin metabolites determined in eight female mice 12 h after injection in four consecutive days; Table S5: Average concentrations of doxorubicin metabolites determined in eight male mice 12 h after injection in four consecutive days.

## Sample Availability

Correspondence and requests for materials should be addressed to R.F. and I.V.V.
