# Supplementary material for: High-Throughput Method for the Simultaneous Determination of Doxorubicin Metabolites in Rat Urine after Treatment with Different Drug Nanoformulations
Source: Molecules. 2022 Feb 9;27(4):1177. doi: 10.3390/molecules27041177 (PMC8877250; doi:10.3390/molecules27041177)
Supplement: Supplementary file 1 [file molecules-27-01177-s001.zip › molecules-1530412-supplementary.pdf]

Supplementary Materials

High-Throughput Method for the Simultaneous Determination of Doxorubicin Metabolites in Rat Urine after Treatment with Different Drug Nanoformulations

Lara Zorić [1], Nikša Drinković [2], Vedran Micek [3], Leo Frkanec [4], Akif Emre Türeli [5], Nazende Günday-Türeli [5], Ivana Vinković Vrček [3,*] and Ruža Frkanec [6,*]

[1] Faculty of Pharmacy and Biochemistry, University of Zagreb, Ante Kovačića 1, 10000 Zagreb, Croatia; larazoric488@gmail.com
[2] Poliklinika Prof. Nikša Drinković, Boškovićeva ul. 15, 10000 Zagreb, Croatia; ndrink@gmail.com
[3] Institute for Medical Research and Occupational Health, Ksaverska cesta 2, 10000 Zagreb, Croatia; vmicek@imi.hr
[4] Rudjer Boskovic Institute, Bijenička cesta 54, 10000 Zagreb, Croatia; frkanec@irb.hr
[5] MyBiotech GmbH, Industriestraße 1B, 66802 Überherrn, Germany; e.tuereli@mybiotech.de (A.E.T.); n.guenday-tuereli@mybiotech.de (N.G.-T.)
[6] University of Zagreb, Centre for Research and Knowledge Transfer in Biotechnology, Rockefellerova 10, 10000 Zagreb, Croatia
* Correspondence: ivinkovic@imi.hr (I.V.V.); rfrkanec@unizg.hr (R.F.)

The linearity of the method

The samples were injected three times, and the mean value of the chromatogram area were calculated. The results are presented graphically (Figures S1-S3) and tabularly (Table S1) for each metabolite. The correlation coefficients are calculated.

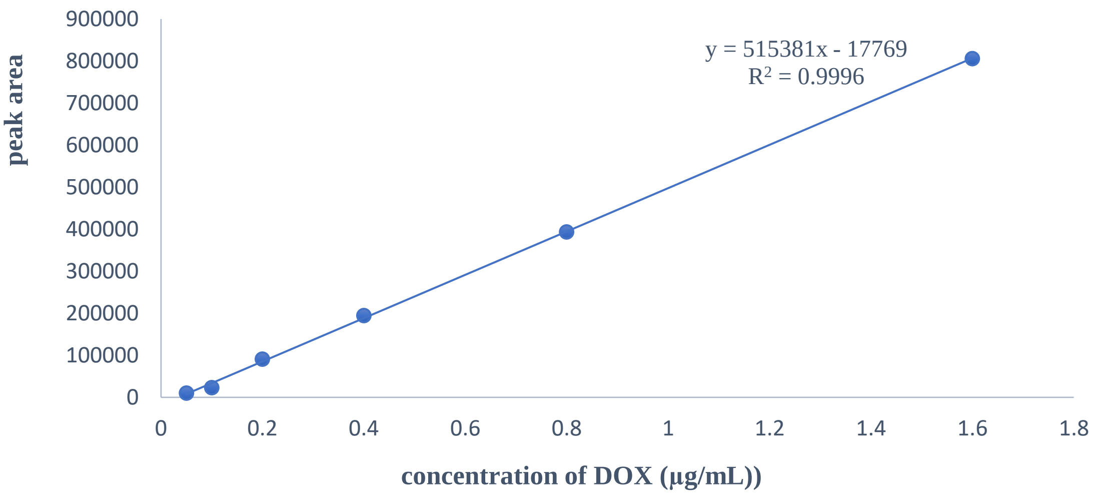

Figure S1. Calibration curve for DOXon.

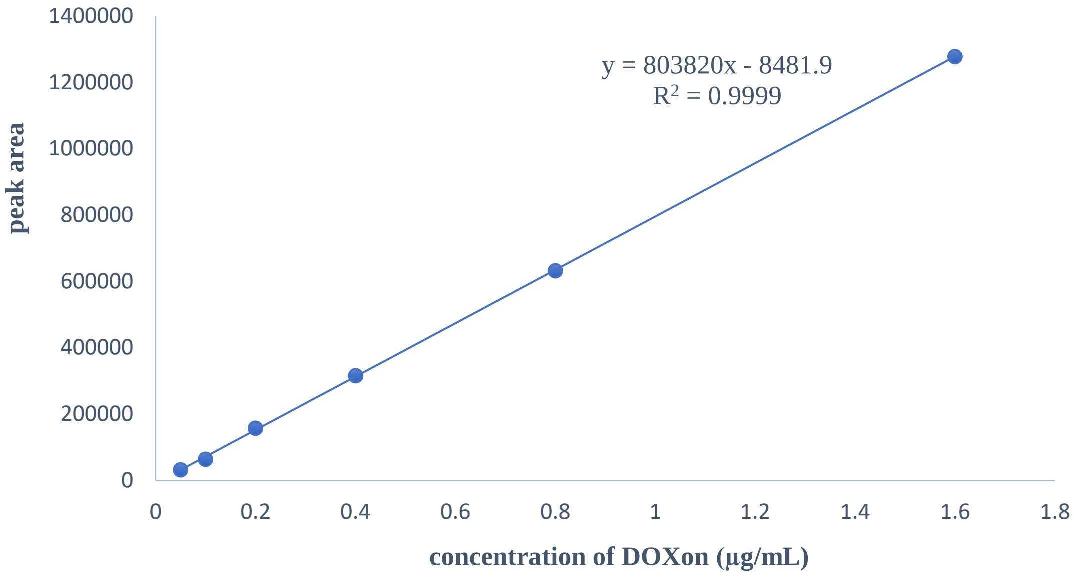

Figure S2. Calibration curve for DOXon.

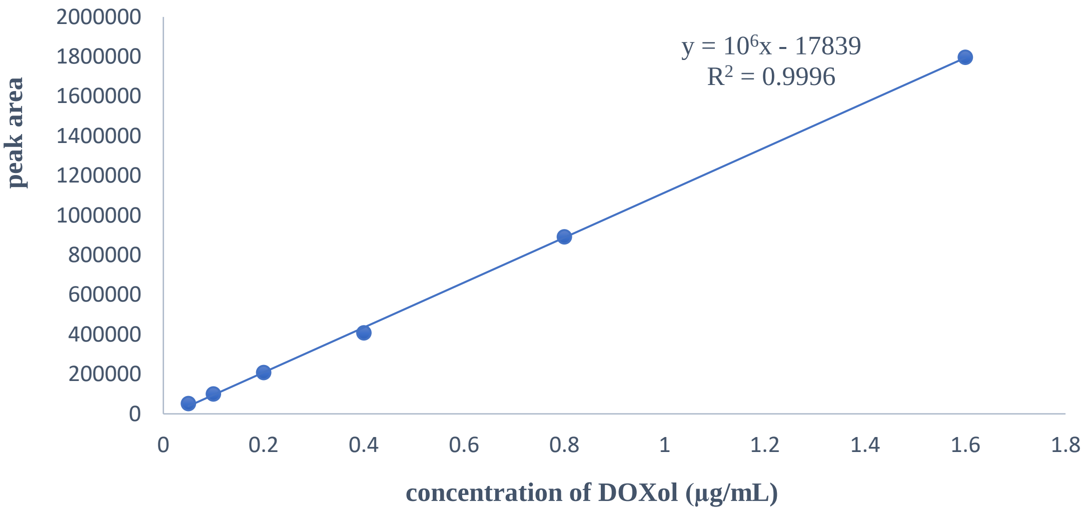

Figure S3. Calibration curve for DOXol.

Table S1. Parameters of linearity.

Metabolite	Linearity area (μg/mL)	Equation of a Line	Factor of regression (R^2)
**DOX**	0,05 – 1,60	y = 515381x - 17769	0,9996
**DOXol**	0,05 – 1,60	y = 10^6 x - 17839	0,9996
**DOXon**	0,05 – 1,60	y = 803820x - 848	0,9999

Table S2. Concentration of doxorubicin (DOX) and its metabolites doxorubicinol (DOXol) and doxorubiconone (DOXon) in urine of female Wistar rats after each of 4 intraperitoneal applications of DOX-IN, DOX-MY and DOX-PLGA.

Exp group	Exp animal	Metabolites (μg/mL)											
		**Doxorubicinol**				**Doxorubicin**				**Doxorubicinon**			
		**1**	**2**	**3**	**4**	**1**	**2**	**3**	**4**	**1**	**2**	**3**	**4**
F DOX-IN	1	0,22	0,41	0,17	0,27	0,15	1,54	0,32	0,94	0,00	0,01	0,00	0,00
	2	0,23	0,20	0,21	0,24	0,29	0,82	1,22	1,02	0,00	0,00	0,00	0,00
	3	0,15	0,35	0,33	0,34	0,21	1,29	1,28	1,25	0,00	0,02	0,00	0,00
	4	0,20	0,54	0,14	0,42	0,35	2,83	0,58	1,66	0,00	0,01	0,00	0,00
	5	0,09	0,22	0,12	0,22	0,10	1,29	0,63	1,34	0,00	0,00	0,00	0,00
	6	0,11	0,15	0,11	0,26	0,17	0,90	0,65	1,26	0,00	0,01	0,00	0,00
	7	0,24	0,19	0,11	0,31	0,65	0,81	0,63	1,81	0,00	0,00	0,00	0,00
	8	0,21	0,32	0,33	0,27	0,87	1,06	1,18	1,54	0,02	0,00	0,00	0,00
F DOX-PLGA	1	0,08	0,15	0,12	0,00	0,48	0,84	0,81	1,96	0,00	0,03	0,00	0,00
	2	0,15	0,16	0,11	0,29	0,62	0,90	0,88	1,14	0,00	0,02	0,00	0,00
	3	0,19		0,28	0,25	0,63		1,14	0,98	0,00		0,00	0,00
	4	0,17	0,22		0,00	0,52	1,10		1,70	0,00	0,00		0,00
	5	0,04	0,14	0,09	0,24	0,26	0,72	0,97	1,75	0,00	0,03	0,09	0,00
	6	0,14	0,12	0,17	0,36	0,65	0,53	1,17	1,62	0,00	0,00	0,05	0,00
	7	0,16	0,10	0,12	0,36	0,75	0,40	0,91	0,68	0,00	0,00	0,00	0,00
	8		0,39	0,21	0,08		2,02	0,81	0,76		0,08	0,00	0,00
F DOX-MY	1	0,04	0,24	0,04		0,13	0,07	0,23		0,00	0,03	0,00	
	2	0,04	0,16	0,07	0,25	0,27	0,13	0,64	1,48	0,00	0,02	0,00	0,00
	3	0,00	0,12	0,05	0,08	0,03	0,25	0,49	0,39	0,00	0,01	0,00	0,00
	4	0,10	0,09	0,06	0,10	0,63	0,36	0,52	0,73	0,00	0,02	0,00	0,00
	5	0,00	0,07	0,05	0,12	0,06	0,30	0,32	1,24	0,00	0,01	0,00	0,00
	6	0,04	0,03	0,05	0,22	0,06	0,14	0,53	0,76	0,00	0,00	0,00	0,00
	7	0,03	0,09		0,10	0,11	0,34		0,55	0,00	0,01		0,00
	8	0,11	0,09	0,08	0,17	0,34	0,47	1,13	1,35	0,00	0,00	0,00	0,00

Table S3. Concentration of doxorubicin (DOX) and its metabolites doxorubicinol (DOXol) and doxorubiconone (DOXon) in urine of male Wistar rats after each of 4 intraperitoneal applications of DOX-IN, DOX-MY and DOX-PLGA.

Exp group	Exp. animal	Metabolites (μg/mL)											
		Doxorubicinol				Doxorubicin				Doxorubicinon			
		1	2	3	4	1	2	3	4	1	2	3	4
M DOX-IN	1	0,00	0,48	0,41	0,00	0,48	2,81	1,09	5,39	0,00	0,06	0,00	0,00
	2	0,33	0,65	0,36	0,00	0,86	2,64	1,15	7,07	0,00	0,05	0,00	0,00
	3	1,00	0,54	0,50	1,04	1,55	2,82	1,62	2,87	0,04	0,04	0,00	0,00
	4	2,97	0,38	0,58	1,47	2,91	1,79	1,98	4,97	0,14	0,01	0,00	0,00
	5	0,34	1,31	0,78	1,34	0,81	2,33	1,60	19,53	0,06	0,01	0,00	0,00
	6	0,56	1,07	0,80	0,52	1,89	2,34	3,45	9,79	0,03	0,05	0,00	0,00
	7	0,56	0,53	0,89	0,29	0,61	3,88	3,94	5,40	0,00	0,00	0,00	0,00
	8	0,44	0,62	0,67	0,59	0,46	4,14	3,56	2,37	0,00	0,05	0,00	0,00
M DOX-PLGA	1	1,83	1,08		0,35	0,17	1,37		1,90	0,06	0,08		0,00
	2	1,14	1,64	0,27	0,24	0,97	1,13	1,43	1,32	0,04	0,02	0,00	0,00
	3	0,55	0,42	0,34	0,32	2,23	0,99	1,72	1,91	0,04	0,01	0,00	0,00
	4	0,28	0,50	0,26	0,29	1,87	1,26	1,23	1,48	0,00	0,00	0,00	0,00
	5	0,31	0,29	0,27	0,24	1,84	1,33	1,36	1,22	0,00	0,02	0,00	0,00
	6	0,64	0,37	0,26	0,23	2,57	1,49	1,26	0,92	0,04	0,04	0,00	0,00
	7	0,31	0,30	0,05	0,38	1,66	1,32	0,17	1,72	0,03	0,00	2,56	0,00
	8	0,34	0,76	0,32	0,50	1,71	1,60	1,83	1,73	0,03	0,04	0,07	0,00
M DOX-MY	1	0,00	0,16	0,15	0,22	0,27	0,45	1,47	1,13	0,00	0,00	0,00	0,00
	2	0,00	0,17	0,23	0,15	0,32	1,52	1,14	0,90	0,00	0,03	0,00	0,00
	3	0,11	0,22	3,24	0,21	0,57	1,54	5,27	1,19	0,00	0,03	0,00	0,00
	4	0,00	0,16	0,27	0,32	0,39	0,80	1,00	1,55	0,00	0,01	0,00	0,00
	5	0,33	0,11	0,15	0,41	0,65	0,86	1,98	2,40	0,00	0,02	0,00	0,00
	6	0,21	0,15	0,23	0,40	1,75	0,58	1,77	2,38	0,01	0,00	0,06	0,00
	7	0,13	0,15	0,33	0,49	1,13	1,03	1,89	1,48	0,00	0,02	0,00	0,00
	8	0,11	0,17	0,19	0,46	0,51	1,28	1,39	2,00	0,01	0,02	0,05	0,00

Table S4. Average concentrations of doxorubicin metabolites determined in eight female mice 12 hours after injection in four consecutive days.

Exp. group		Metabolites (μg/mL)											
		Doxorubicinol				Doxorubicin				Doxorubicinone			
		1	2	3	4	1	2	3	4	1	2	3	4
F DOX	x_{avg}:	0,18	0,30	0,19	0,30	0,35	1,32	0,81	1,35	0,00	0,01	0,00	0,00
	S_d:	0,06	0,13	0,09	0,06	0,27	0,66	0,36	0,30	0,01	0,01	0,00	0,00
F PD	x_{avg}:	0,13	0,18	0,16	0,20	0,56	0,93	0,96	1,32	0,00	0,02	0,02	0,00
	S_d:	0,05	0,10	0,07	0,15	0,16	0,53	0,15	0,49	0,00	0,03	0,04	0,00
F MY	x_{avg}:	0,05	0,11	0,06	0,15	0,20	0,26	0,55	0,93	0,00	0,01	0,00	0,00
	S_d	0,04	0,06	0,01	0,07	0,20	0,14	0,29	0,42	0,00	0,01	0,00	0,00

Table S5. Average concentrations of doxorubicin metabolites determined in eight male mice 12 hours after injection in four consecutive days.

**Exp. group**		**Metabolites (µg/mL)**											
		**Doxorubicinol**				**Doxorubicin**				**Doxorubicinone**			
		**1**	**2**	**3**	**4**	**1**	2	3	4	1	2	3	4
M DOX	x_{avg}:	0,78	0,70	0,62	0,66	1,20	2,84	2,30	7,17	0,03	0,03	0,00	0,00
	S_d:	0,93	0,32	0,19	0,57	0,86	0,80	1,16	5,51	0,05	0,02	0,00	0,00
M PD	x_{avg}:	0,68	0,67	0,25	0,32	1,63	1,31	1,29	1,53	0,03	0,03	0,38	0,00
	S_d:	0,55	0,48	0,09	0,09	0,75	0,19	0,54	0,35	0,02	0,03	0,96	0,00
M MY	x_{avg}:	0,11	0,16	0,60	0,33	0,70	1,01	1,99	1,63	0,00	0,02	0,01	0,00
	S_d	0,12	0,03	1,07	0,13	0,50	0,41	1,37	0,57	0,00	0,01	0,03	0,00
